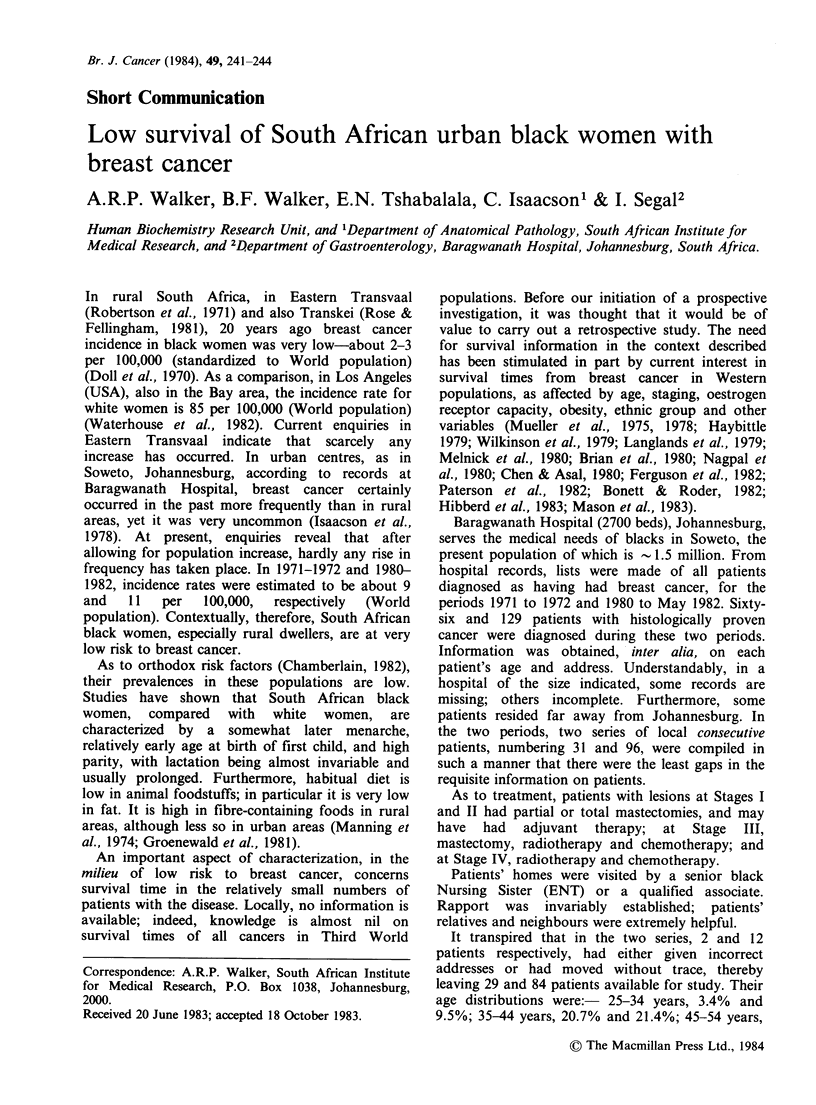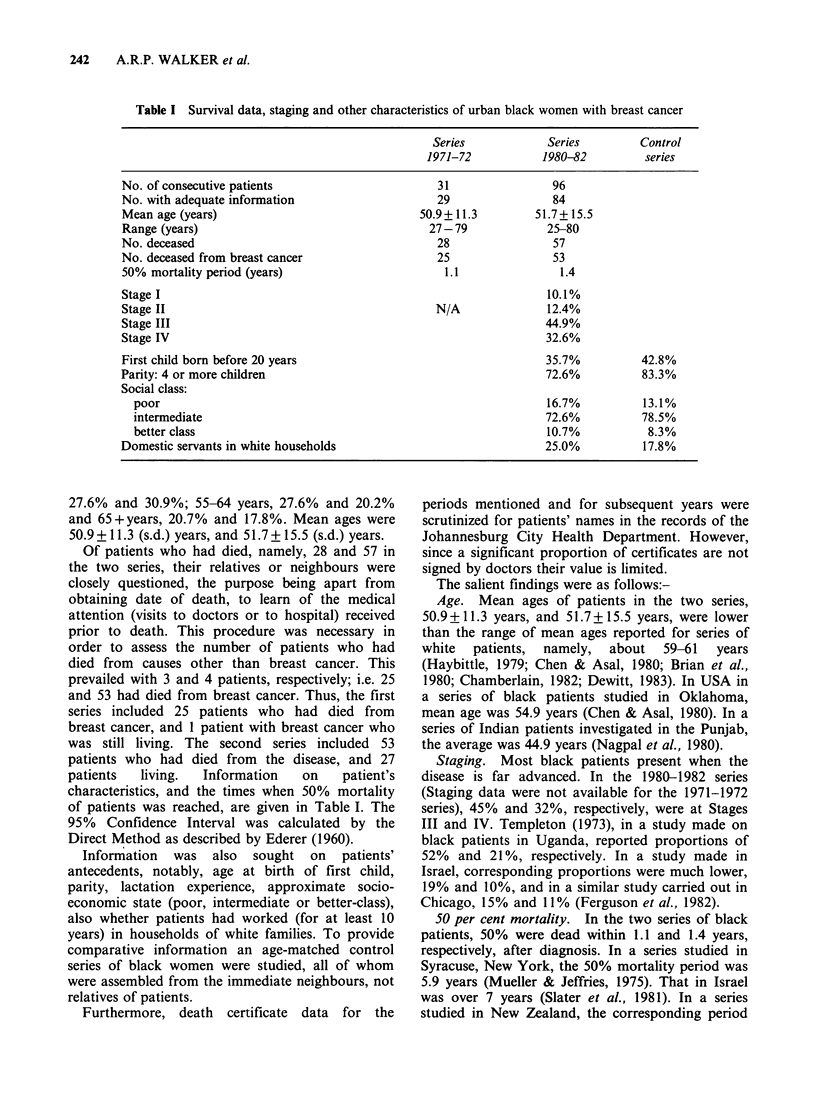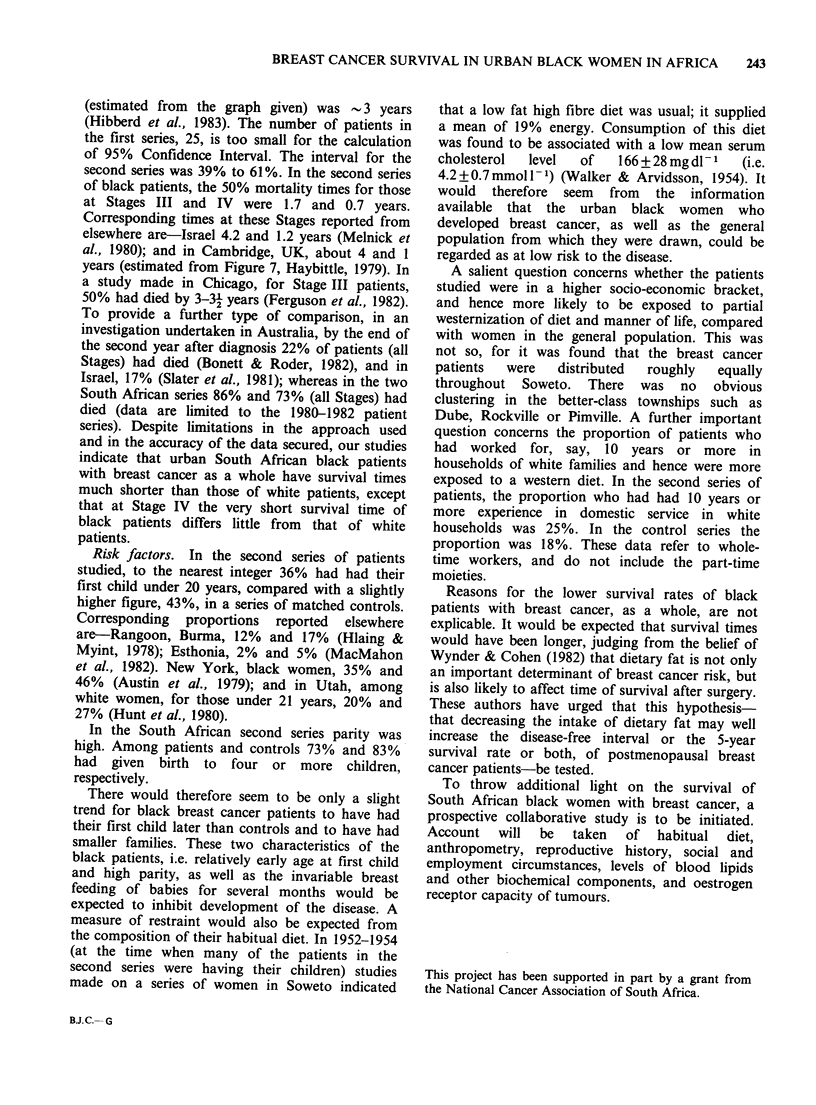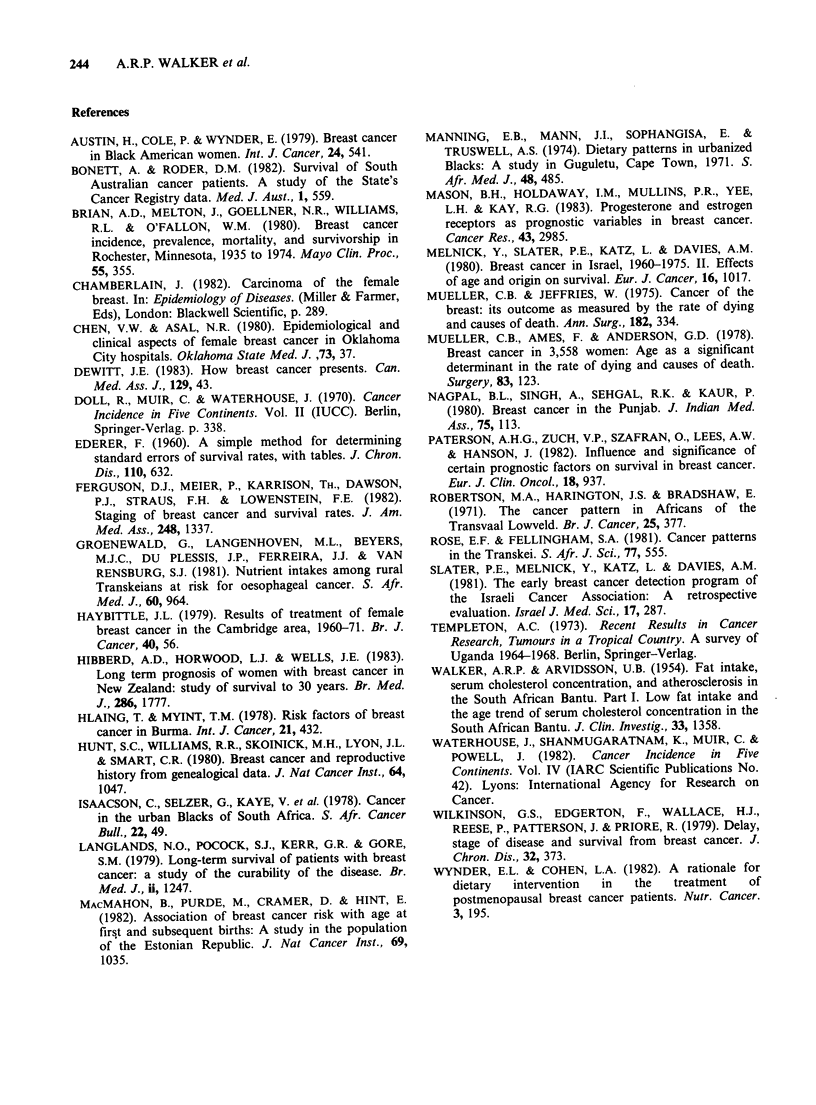# Low survival of South African urban black women with breast cancer.

**DOI:** 10.1038/bjc.1984.37

**Published:** 1984-02

**Authors:** A. R. Walker, B. F. Walker, E. N. Tshabalala, C. Isaacson, I. Segal


*Br. J. Cancer* (1984), **49**, 241–244

### Short Communication

### Low survival of South African urban black women with breast cancer

A.R.P. Walker, B.F. Walker, E.N. Tshabalala, C. Isaacson[1] & I. Segal[2]

*Human Biochemistry Research Unit, and [1]Department of Anatomical Pathology, South African Institute for Medical Research, and [2]Department of Gastroenterology, Baragwanath Hospital, Johannesburg, South Africa.*

In rural South Africa, in Eastern Transvaal (Robertson *et al.*, 1971) and also Transkei (Rose & Fellingham, 1981), 20 years ago breast cancer incidence in black women was very low—about 2–3 per 100,000 (standardized to World population) (Doll *et al.*, 1970). As a comparison, in Los Angeles (USA), also in the Bay area, the incidence rate for white women is 85 per 100,000 (World population) (Waterhouse *et al.*, 1982). Current enquiries in Eastern Transvaal indicate that scarcely any increase has occurred. In urban centres, as in Soweto, Johannesburg, according to records at Baragwanath Hospital, breast cancer certainly occurred in the past more frequently than in rural areas, yet it was very uncommon (Isaacson *et al.*, 1978). At present, enquiries reveal that after allowing for population increase, hardly any rise in frequency has taken place. In 1971–1972 and 1980–1982, incidence rates were estimated to be about 9 and 11 per 100,000, respectively (World population). Contextually, therefore, South African black women, especially rural dwellers, are at very low risk to breast cancer.

As to orthodox risk factors (Chamberlain, 1982), their prevalences in these populations are low. Studies have shown that South African black women, compared with white women, are characterized by a somewhat later menarche, relatively early age at birth of first child, and high parity, with lactation being almost invariable and usually prolonged. Furthermore, habitual diet is low in animal foodstuffs; in particular it is very low in fat. It is high in fibre-containing foods in rural areas, although less so in urban areas (Manning *et al.*, 1974; Groenewald *et al.*, 1981).

An important aspect of characterization, in the *milieu* of low risk to breast cancer, concerns survival time in the relatively small numbers of patients with the disease. Locally, no information is available; indeed, knowledge is almost nil on survival times of all cancers in Third World populations. Before our initiation of a prospective investigation, it was thought that it would be of value to carry out a retrospective study. The need for survival information in the context described has been stimulated in part by current interest in survival times from breast cancer in Western populations, as affected by age, staging, oestrogen receptor capacity, obesity, ethnic group and other variables (Mueller *et al.*, 1975, 1978; Haybittle 1979; Wilkinson *et al.*, 1979; Langlands *et al.*, 1979; Melnick *et al.*, 1980; Brian *et al.*, 1980; Nagpal *et al.*, 1980; Chen & Asal, 1980; Ferguson *et al.*, 1982; Paterson *et al.*, 1982; Bonett & Roder, 1982; Hibberd *et al.*, 1983; Mason *et al.*, 1983).

Baragwanath Hospital (2700 beds), Johannesburg, serves the medical needs of blacks in Soweto, the present population of which is $\sim$1.5 million. From hospital records, lists were made of all patients diagnosed as having had breast cancer, for the periods 1971 to 1972 and 1980 to May 1982. Sixty-six and 129 patients with histologically proven cancer were diagnosed during these two periods. Information was obtained, *inter alia*, on each patient's age and address. Understandably, in a hospital of the size indicated, some records are missing; others incomplete. Furthermore, some patients resided far away from Johannesburg. In the two periods, two series of local *consecutive* patients, numbering 31 and 96, were compiled in such a manner that there were the least gaps in the requisite information on patients.

As to treatment, patients with lesions at Stages I and II had partial or total mastectomies, and may have had adjuvant therapy; at Stage III, mastectomy, radiotherapy and chemotherapy; and at Stage IV, radiotherapy and chemotherapy.

Patients' homes were visited by a senior black Nursing Sister (ENT) or a qualified associate. Rapport was invariably established; patients' relatives and neighbours were extremely helpful.

It transpired that in the two series, 2 and 12 patients respectively, had either given incorrect addresses or had moved without trace, thereby leaving 29 and 84 patients available for study. Their age distributions were:— 25–34 years, 3.4% and 9.5%; 35–44 years, 20.7% and 21.4%; 45–54 years,

Correspondence: A.R.P. Walker, South African Institute for Medical Research, P.O. Box 1038, Johannesburg, 2000.
Received 20 June 1983; accepted 18 October 1983.

© The Macmillan Press Ltd., 1984

**Table I** Survival data, staging and other characteristics of urban black women with breast cancer

| | *Series 1971–72* | *Series 1980–82* | *Control series* |
|---|---|---|---|
| No. of consecutive patients | 31 | 96 | |
| No. with adequate information | 29 | 84 | |
| Mean age (years) | 50.9±11.3 | 51.7±15.5 | |
| Range (years) | 27–79 | 25–80 | |
| No. deceased | 28 | 57 | |
| No. deceased from breast cancer | 25 | 53 | |
| 50% mortality period (years) | 1.1 | 1.4 | |
| Stage I | | 10.1% | |
| Stage II | N/A | 12.4% | |
| Stage III | | 44.9% | |
| Stage IV | | 32.6% | |
| First child born before 20 years | | 35.7% | 42.8% |
| Parity: 4 or more children | | 72.6% | 83.3% |
| Social class: | | | |
| poor | | 16.7% | 13.1% |
| intermediate | | 72.6% | 78.5% |
| better class | | 10.7% | 8.3% |
| Domestic servants in white households | | 25.0% | 17.8% |

27.6% and 30.9%; 55–64 years, 27.6% and 20.2% and 65+years, 20.7% and 17.8%. Mean ages were 50.9±11.3 (s.d.) years, and 51.7±15.5 (s.d.) years.

Of patients who had died, namely, 28 and 57 in the two series, their relatives or neighbours were closely questioned, the purpose being apart from obtaining date of death, to learn of the medical attention (visits to doctors or to hospital) received prior to death. This procedure was necessary in order to assess the number of patients who had died from causes other than breast cancer. This prevailed with 3 and 4 patients, respectively; i.e. 25 and 53 had died from breast cancer. Thus, the first series included 25 patients who had died from breast cancer, and 1 patient with breast cancer who was still living. The second series included 53 patients who had died from the disease, and 27 patients living. Information on patient's characteristics, and the times when 50% mortality of patients was reached, are given in Table I. The 95% Confidence Interval was calculated by the Direct Method as described by Ederer (1960).

Information was also sought on patients' antecedents, notably, age at birth of first child, parity, lactation experience, approximate socio-economic state (poor, intermediate or better-class), also whether patients had worked (for at least 10 years) in households of white families. To provide comparative information an age-matched control series of black women were studied, all of whom were assembled from the immediate neighbours, not relatives of patients.

Furthermore, death certificate data for the periods mentioned and for subsequent years were scrutinized for patients' names in the records of the Johannesburg City Health Department. However, since a significant proportion of certificates are not signed by doctors their value is limited.

The salient findings were as follows:–

*Age.* Mean ages of patients in the two series, 50.9±11.3 years, and 51.7±15.5 years, were lower than the range of mean ages reported for series of white patients, namely, about 59–61 years (Haybittle, 1979; Chen & Asal, 1980; Brian *et al.*, 1980; Chamberlain, 1982; Dewitt, 1983). In USA in a series of black patients studied in Oklahoma, mean age was 54.9 years (Chen & Asal, 1980). In a series of Indian patients investigated in the Punjab, the average was 44.9 years (Nagpal *et al.*, 1980).

*Staging.* Most black patients present when the disease is far advanced. In the 1980–1982 series (Staging data were not available for the 1971–1972 series), 45% and 32%, respectively, were at Stages III and IV. Templeton (1973), in a study made on black patients in Uganda, reported proportions of 52% and 21%, respectively. In a study made in Israel, corresponding proportions were much lower, 19% and 10%, and in a similar study carried out in Chicago, 15% and 11% (Ferguson *et al.*, 1982).

*50 per cent mortality.* In the two series of black patients, 50% were dead within 1.1 and 1.4 years, respectively, after diagnosis. In a series studied in Syracuse, New York, the 50% mortality period was 5.9 years (Mueller & Jeffries, 1975). That in Israel was over 7 years (Slater *et al.*, 1981). In a series studied in New Zealand, the corresponding period

(estimated from the graph given) was ~3 years (Hibberd *et al.*, 1983). The number of patients in the first series, 25, is too small for the calculation of 95% Confidence Interval. The interval for the second series was 39% to 61%. In the second series of black patients, the 50% mortality times for those at Stages III and IV were 1.7 and 0.7 years. Corresponding times at these Stages reported from elsewhere are—Israel 4.2 and 1.2 years (Melnick *et al.*, 1980); and in Cambridge, UK, about 4 and 1 years (estimated from Figure 7, Haybittle, 1979). In a study made in Chicago, for Stage III patients, 50% had died by 3–3½ years (Ferguson *et al.*, 1982). To provide a further type of comparison, in an investigation undertaken in Australia, by the end of the second year after diagnosis 22% of patients (all Stages) had died (Bonett & Roder, 1982), and in Israel, 17% (Slater *et al.*, 1981); whereas in the two South African series 86% and 73% (all Stages) had died (data are limited to the 1980–1982 patient series). Despite limitations in the approach used and in the accuracy of the data secured, our studies indicate that urban South African black patients with breast cancer as a whole have survival times much shorter than those of white patients, except that at Stage IV the very short survival time of black patients differs little from that of white patients.

*Risk factors.* In the second series of patients studied, to the nearest integer 36% had had their first child under 20 years, compared with a slightly higher figure, 43%, in a series of matched controls. Corresponding proportions reported elsewhere are—Rangoon, Burma, 12% and 17% (Hlaing & Myint, 1978); Esthonia, 2% and 5% (MacMahon *et al.*, 1982). New York, black women, 35% and 46% (Austin *et al.*, 1979); and in Utah, among white women, for those under 21 years, 20% and 27% (Hunt *et al.*, 1980).

In the South African second series parity was high. Among patients and controls 73% and 83% had given birth to four or more children, respectively.

There would therefore seem to be only a slight trend for black breast cancer patients to have had their first child later than controls and to have had smaller families. These two characteristics of the black patients, i.e. relatively early age at first child and high parity, as well as the invariable breast feeding of babies for several months would be expected to inhibit development of the disease. A measure of restraint would also be expected from the composition of their habitual diet. In 1952–1954 (at the time when many of the patients in the second series were having their children) studies made on a series of women in Soweto indicated that a low fat high fibre diet was usual; it supplied a mean of 19% energy. Consumption of this diet was found to be associated with a low mean serum cholesterol level of $166 \pm 28\,\mathrm{mg\,dl^{-1}}$ (i.e. $4.2 \pm 0.7\,\mathrm{mmol\,l^{-1}}$) (Walker & Arvidsson, 1954). It would therefore seem from the information available that the urban black women who developed breast cancer, as well as the general population from which they were drawn, could be regarded as at low risk to the disease.

A salient question concerns whether the patients studied were in a higher socio-economic bracket, and hence more likely to be exposed to partial westernization of diet and manner of life, compared with women in the general population. This was not so, for it was found that the breast cancer patients were distributed roughly equally throughout Soweto. There was no obvious clustering in the better-class townships such as Dube, Rockville or Pimville. A further important question concerns the proportion of patients who had worked for, say, 10 years or more in households of white families and hence were more exposed to a western diet. In the second series of patients, the proportion who had had 10 years or more experience in domestic service in white households was 25%. In the control series the proportion was 18%. These data refer to whole-time workers, and do not include the part-time moieties.

Reasons for the lower survival rates of black patients with breast cancer, as a whole, are not explicable. It would be expected that survival times would have been longer, judging from the belief of Wynder & Cohen (1982) that dietary fat is not only an important determinant of breast cancer risk, but is also likely to affect time of survival after surgery. These authors have urged that this hypothesis—that decreasing the intake of dietary fat may well increase the disease-free interval or the 5-year survival rate or both, of postmenopausal breast cancer patients—be tested.

To throw additional light on the survival of South African black women with breast cancer, a prospective collaborative study is to be initiated. Account will be taken of habitual diet, anthropometry, reproductive history, social and employment circumstances, levels of blood lipids and other biochemical components, and oestrogen receptor capacity of tumours.

This project has been supported in part by a grant from the National Cancer Association of South Africa.

**References**

AUSTIN, H., COLE, P. & WYNDER, E. (1979). Breast cancer in Black American women. *Int. J. Cancer*, **24**, 541.

BONETT, A. & RODER, D.M. (1982). Survival of South Australian cancer patients. A study of the State's Cancer Registry data. *Med. J. Aust.*, **1**, 559.

BRIAN, A.D., MELTON, J., GOELLNER, N.R., WILLIAMS, R.L. & O'FALLON, W.M. (1980). Breast cancer incidence, prevalence, mortality, and survivorship in Rochester, Minnesota, 1935 to 1974. *Mayo Clin. Proc.*, **55**, 355.

CHAMBERLAIN, J. (1982). Carcinoma of the female breast. In: *Epidemiology of Diseases*. (Miller & Farmer, Eds), London: Blackwell Scientific, p. 289.

CHEN, V.W. & ASAL, N.R. (1980). Epidemiological and clinical aspects of female breast cancer in Oklahoma City hospitals. *Oklahoma State Med. J.* ,**73**, 37.

DEWITT, J.E. (1983). How breast cancer presents. *Can. Med. Ass. J.*, **129**, 43.

DOLL, R., MUIR, C. & WATERHOUSE, J. (1970). *Cancer Incidence in Five Continents*. Vol. II (IUCC). Berlin, Springer-Verlag. p. 338.

EDERER, F. (1960). A simple method for determining standard errors of survival rates, with tables. *J. Chron. Dis.*, **110**, 632.

FERGUSON, D.J., MEIER, P., KARRISON, TH., DAWSON, P.J., STRAUS, F.H. & LOWENSTEIN, F.E. (1982). Staging of breast cancer and survival rates. *J. Am. Med. Ass.*, **248**, 1337.

GROENEWALD, G., LANGENHOVEN, M.L., BEYERS, M.J.C., DU PLESSIS, J.P., FERREIRA, J.J. & VAN RENSBURG, S.J. (1981). Nutrient intakes among rural Transkeians at risk for oesophageal cancer. *S. Afr. Med. J.*, **60**, 964.

HAYBITTLE, J.L. (1979). Results of treatment of female breast cancer in the Cambridge area, 1960–71. *Br. J. Cancer*, **40**, 56.

HIBBERD, A.D., HORWOOD, L.J. & WELLS, J.E. (1983). Long term prognosis of women with breast cancer in New Zealand: study of survival to 30 years. *Br. Med. J.*, **286**, 1777.

HLAING, T. & MYINT, T.M. (1978). Risk factors of breast cancer in Burma. *Int. J. Cancer*, **21**, 432.

HUNT, S.C., WILLIAMS, R.R., SKOINICK, M.H., LYON, J.L. & SMART, C.R. (1980). Breast cancer and reproductive history from genealogical data. *J. Nat Cancer Inst.*, **64**, 1047.

ISAACSON, C., SELZER, G., KAYE, V. *et al.* (1978). Cancer in the urban Blacks of South Africa. *S. Afr. Cancer Bull.*, **22**, 49.

LANGLANDS, N.O., POCOCK, S.J., KERR, G.R. & GORE, S.M. (1979). Long-term survival of patients with breast cancer: a study of the curability of the disease. *Br. Med. J.*, **ii**, 1247.

MACMAHON, B., PURDE, M., CRAMER, D. & HINT, E. (1982). Association of breast cancer risk with age at first and subsequent births: A study in the population of the Estonian Republic. *J. Nat Cancer Inst.*, **69**, 1035.

MANNING, E.B., MANN, J.I., SOPHANGISA, E. & TRUSWELL, A.S. (1974). Dietary patterns in urbanized Blacks: A study in Guguletu, Cape Town, 1971. *S. Afr. Med. J.*, **48**, 485.

MASON, B.H., HOLDAWAY, I.M., MULLINS, P.R., YEE, L.H. & KAY, R.G. (1983). Progesterone and estrogen receptors as prognostic variables in breast cancer. *Cancer Res.*, **43**, 2985.

MELNICK, Y., SLATER, P.E., KATZ, L. & DAVIES, A.M. (1980). Breast cancer in Israel, 1960–1975. II. Effects of age and origin on survival. *Eur. J. Cancer*, **16**, 1017.

MUELLER, C.B. & JEFFRIES, W. (1975). Cancer of the breast: its outcome as measured by the rate of dying and causes of death. *Ann. Surg.*, **182**, 334.

MUELLER, C.B., AMES, F. & ANDERSON, G.D. (1978). Breast cancer in 3,558 women: Age as a significant determinant in the rate of dying and causes of death. *Surgery*, **83**, 123.

NAGPAL, B.L., SINGH, A., SEHGAL, R.K. & KAUR, P. (1980). Breast cancer in the Punjab. *J. Indian Med. Ass.*, **75**, 113.

PATERSON, A.H.G., ZUCH, V.P., SZAFRAN, O., LEES, A.W. & HANSON, J. (1982). Influence and significance of certain prognostic factors on survival in breast cancer. *Eur. J. Clin. Oncol.*, **18**, 937.

ROBERTSON, M.A., HARINGTON, J.S. & BRADSHAW, E. (1971). The cancer pattern in Africans of the Transvaal Lowveld. *Br. J. Cancer*, **25**, 377.

ROSE, E.F. & FELLINGHAM, S.A. (1981). Cancer patterns in the Transkei. *S. Afr. J. Sci.*, **77**, 555.

SLATER, P.E., MELNICK, Y., KATZ, L. & DAVIES, A.M. (1981). The early breast cancer detection program of the Israeli Cancer Association: A retrospective evaluation. *Israel J. Med. Sci.*, **17**, 287.

TEMPLETON, A.C. (1973). *Recent Results in Cancer Research, Tumours in a Tropical Country*. A survey of Uganda 1964–1968. Berlin, Springer–Verlag.

WALKER, A.R.P. & ARVIDSSON, U.B. (1954). Fat intake, serum cholesterol concentration, and atherosclerosis in the South African Bantu. Part I. Low fat intake and the age trend of serum cholesterol concentration in the South African Bantu. *J. Clin. Investig.*, **33**, 1358.

WATERHOUSE, J., SHANMUGARATNAM, K., MUIR, C. & POWELL, J. (1982). *Cancer Incidence in Five Continents*. Vol. IV (IARC Scientific Publications No. 42). Lyons: International Agency for Research on Cancer.

WILKINSON, G.S., EDGERTON, F., WALLACE, H.J., REESE, P., PATTERSON, J. & PRIORE, R. (1979). Delay, stage of disease and survival from breast cancer. *J. Chron. Dis.*, **32**, 373.

WYNDER, E.L. & COHEN, L.A. (1982). A rationale for dietary intervention in the treatment of postmenopausal breast cancer patients. *Nutr. Cancer*. **3**, 195.